# The Prognostic Significance of the Metabolic Score for Insulin Resistance and Subclinical Myocardial Injury for Cardiovascular Mortality in the General Population

**DOI:** 10.3390/jcm15031141

**Published:** 2026-02-02

**Authors:** Patrick Cheon, Shannon O’Connor, Saeid Mirzai, Mohamed A. Mostafa, Chuka B. Ononye, Elsayed Z. Soliman, Richard Kazibwe

**Affiliations:** 1 School of Medicine, Winston-Salem Campus, Wake Forest University, Winston-Salem, NC 27101, USA; patrick.cheon@wfusm.edu; 2 Department of Internal Medicine, Atrium Health Wake Forest Baptist, Winston-Salem, NC 27103, USA; shannon.oconnor@advocatehealth.org (S.O.); mohamed.mostafa@advocatehealth.org (M.A.M.); chuka.ononye@advocatehealth.org (C.B.O.); richard.kazibwe@advocatehealth.org (R.K.); 3 Department of Cardiovascular Medicine, Atrium Health Wake Forest Baptist, Winston-Salem, NC 27157, USA; saeid.mirzai@advocatehealth.org

**Keywords:** Metabolic Score for Insulin Resistance, subclinical myocardial injury, cardiovascular mortality, NHANES III

## Abstract

**Background/Objectives**: The Metabolic Score for Insulin Resistance (METS-IR), a non-insulin-based index of insulin resistance (IR), and subclinical myocardial injury (SCMI), identified by electrocardiogram (ECG), are each associated with cardiovascular disease (CVD). However, their joint impact on mortality remains unclear. We examined the association of the METS-IR with SCMI and evaluated the individual and combined associations of SCMI and IR with cardiovascular mortality. **Methods**: We analyzed adults without baseline CVD from the Third National Health and Nutrition Examination Survey (1988–1994) with mortality follow-up through 31 December 2019. The METS-IR was calculated from fasting glucose, triglycerides, high-density lipoprotein cholesterol, and body mass index and categorized as low (<75th percentile) or high (≥75th percentile). SCMI was defined as a cardiac infarction injury score ≥ 10 on ECG. Multivariable logistic regression assessed associations between the METS-IR and SCMI, and Cox regression estimated cardiovascular mortality risk across SCMI-IR combinations. **Results**: Among 6079 participants, 14.1% had SCMI. Higher METS-IR values were associated with greater SCMI odds (OR (95% CI): 1.58 (1.31–1.90)). Over a median of 18.8 years, 563 (9.1%) cardiovascular deaths occurred. Both SCMI and high IR were individually associated with increased cardiovascular mortality ((HR (95% CI): 1.41 (1.19–1.69) and 1.32 (1.09–1.59), respectively). Participants with both SCMI and high IR had the highest risk (HR 1.92; 95% CI 1.49–2.50) compared with those with neither condition. **Conclusions**: In adults without prior CVD, the METS-IR was positively associated with SCMI. The coexistence of SCMI and high IR identified a subgroup at nearly twofold higher risk of cardiovascular mortality, supporting the combined use of ECG-based injury markers and metabolic indices for cardiovascular risk stratification.

## 1. Introduction

Cardiovascular disease (CVD) remains the leading cause of death globally, responsible for an estimated 19.8 million deaths in 2022—approximately 32% of all deaths worldwide [1]. Therefore, efforts to identify individuals at heightened risk are a cornerstone of strategies for CVD. An important but often overlooked marker of cardiovascular risk is subclinical myocardial injury (SCMI), defined as myocardial damage occurring in individuals without overt CVD. SCMI can be detected via simple surface 12-lead electrocardiogram (ECG) through validated scoring systems such as the cardiac infarction injury score (CIIS) [2,3]. The presence of SCMI has been associated with a 26% higher risk of cardiovascular morality, stressing its relevance in identifying individuals at hidden risk for clinical CVD outcomes [4,5,6].

Insulin resistance (IR) is another major contributor to adverse cardiovascular health. IR promotes metabolic dysfunction and has been associated with more than a twofold higher risk of myocardial infarction and a 62% higher risk of death in previous studies [7,8,9,10]. Gold-standard measures of insulin sensitivity, such as the hyperinsulinemic–euglycemic clamp, are accurate but not feasible for population studies or routine clinical care [8,11]. As a result, non-insulin surrogate markers have been developed. The Metabolic Score for Insulin Resistance (METS-IR), calculated from fasting glucose, triglycerides, high-density lipoprotein cholesterol (HDL-C), and body mass index (BMI), is a simple index that correlates well with clamp-measured insulin sensitivity [8,11,12]. Higher METS-IR values have demonstrated prognostic value for adverse cardiovascular outcomes; across different studies, elevated METS-IR values were independently associated with ischemic heart disease, stroke, heart failure, and cardiovascular mortality [13,14,15,16].

Despite the independent prognostic roles of SCMI and the METS-IR in predicting cardiovascular outcomes, the relationship between these two risk factors has scarcely been studied, and the combined effect of IR and SCMI on cardiovascular (CV) mortality remains unclear. Understanding this effect could reveal novel insights into mechanisms of CVD progression and improve risk stratification. To address this gap, this study utilizes data from the Third National Health and Nutrition Examination Survey (NHANES III) free from clinically apparent CVD at baseline to investigate the independent and joint associations of the METS-IR and SCMI with CV mortality.

## 2. Materials and Methods

### 2.1. Study Design and Population

We analyzed participant data from the NHANES III, a nationally representative survey of the U.S. population conducted between 1988 and 1994 [17,18].

**Figure 1:**
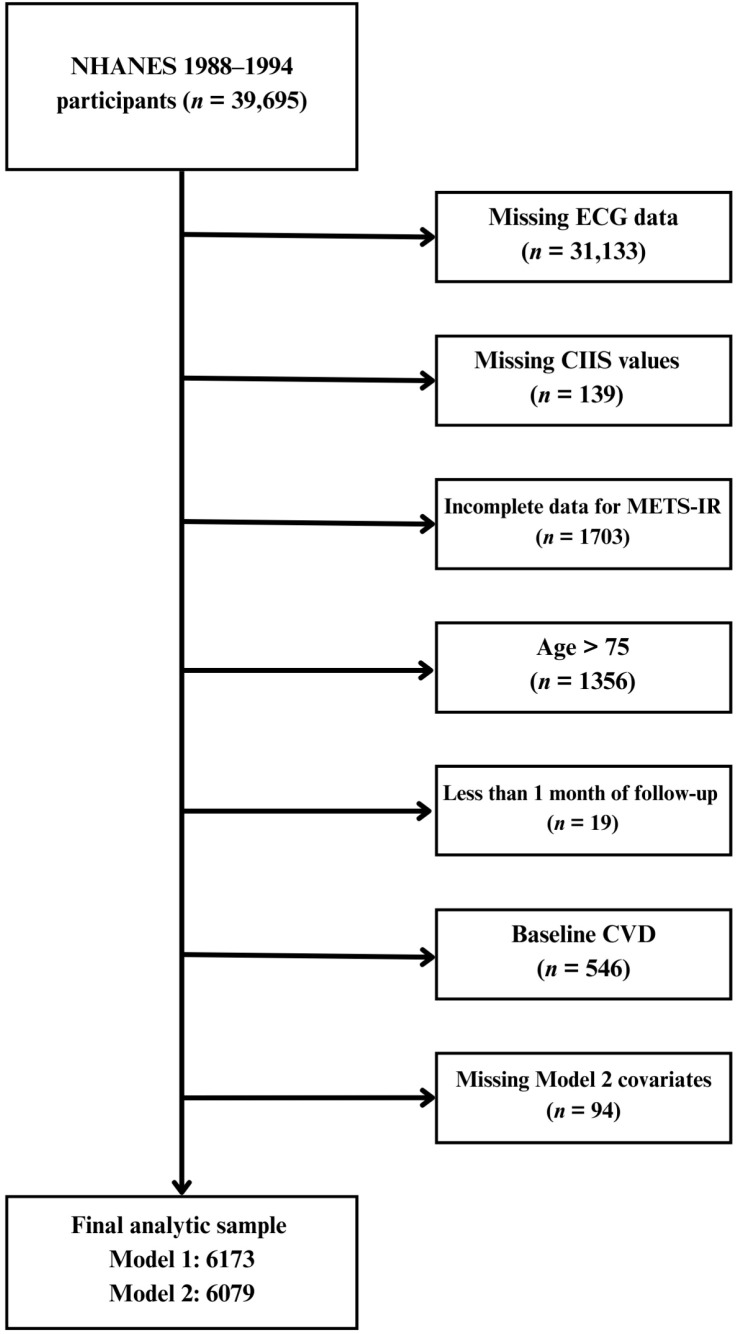
Flowchart of participant selection for the analytic sample. ECG, electrocardiogram; CIIS, cardiac infarction injury score; METS-IR, Metabolic Score for Insulin Resistance; CVD, cardiovascular disease; NHANES, National Health and Nutrition Examination Survey. All data used were publicly available and obtained from the NHANES website (https://wwwn.cdc.gov/nchs/nhanes) (accessed on 20 June 2025).

A total of 8561 participants from the NHANES III with available ECG data were considered for inclusion in this analysis. Participants with missing CIIS values were excluded, leaving 8422 for further evaluation. Additional exclusions were applied to ensure complete data on the variables required to calculate the METS-IR and to adjust for key covariates. Specifically, individuals missing data for triglycerides, HDL-C, glucose, or other essential covariates were removed. Participants aged >75 years (*n* = 1356) and those with less than one month of follow-up (*n* = 19) were also excluded at this stage. After these steps, 6719 participants remained eligible. Finally, individuals with evidence of baseline CVD, defined as self-reported or a prior diagnosis of heart failure, coronary heart disease, angina, myocardial infarction, or stroke, were excluded to restrict the analysis to participants free of established CVD at baseline. This resulted in a preliminary analytic sample of 6173 participants (Figure 1).

### 2.2. Metabolic Score for Insulin Resistance (METS-IR) Calculation

Consistent with previous studies, the METS-IR was calculated using fasting glucose, triglycerides, high-density lipoprotein cholesterol (HDL-C), and body mass index (BMI) [8,11]. The METS-IR was calculated using the following formula:

METS-IR = Ln [(2 ∗ Fasting Plasma Glucose (FPG)) + Triglycerides (TG)] ∗ BMI/Ln (High-Density Lipoprotein Cholesterol (HDL-C)).

As there are no universally established clinical cutoffs for the METS-IR and published thresholds vary by population and outcome, we operationally defined high IR as METS-IR values at or above the 75th percentile of the cohort distribution, and low IR as values below the 75th percentile. This approach aligns with prior percentile-based stratifications used to study METS-IR associations with incident disease and mortality [13,15,16,19].

### 2.3. Subclinical Myocardial Injury (SCMI)

SCMI was defined based on baseline ECG findings. Resting 12-lead ECGs were obtained during the mobile examination center visit using the Marquette MAC 12 system (Marquette Medical Systems, Milwaukee, WI, USA). The ECGs were centrally interpreted, and the cardiac infarction/injury score (CIIS) was calculated using an algorithm that incorporates both discrete and continuous features of Q, R, and T waves and ST segments. SCMI was defined as a CIIS score ≥ 10, a threshold that has been used in prior studies to identify silent or unrecognized myocardial injury [3,4,20,21]. This method provides a reproducible and standardized approach for detecting subclinical cardiac damage in asymptomatic individuals.

### 2.4. Cardiovascular Mortality

The primary outcome of interest was CV mortality. Mortality status and cause of death were attained through linkage of NHANES III data with the National Death Index (NDI) through 31 December 2019. CV mortality was defined using International Classification of Diseases, 10th Revision (ICD-10) codes I00–I78 as the underlying cause of death. The NDI linkage utilized a probabilistic matching algorithm based on multiple identifiers, including name, date of birth, and Social Security number. Person-years of follow-up were calculated from the date of the baseline exam until the date of death or end of follow-up, whichever occurred first.

### 2.5. Covariates

Baseline covariates of age, sex, race/ethnicity, education, income, smoking status, and medication intake were obtained through standardized questionnaires conducted during home interviews. Physical activity was determined according to the frequency of leisure time activity based on the types of activity, frequency, and level of activity. Blood pressure was measured while seated, and up to three measurements were averaged. Diabetes mellitus was defined as a fasting plasma glucose ≥ 126 mg/dL, hemoglobin A1c values ≥ 6.5%, or self-reported use of glucose-lowering medications. Blood samples were collected and analyzed for total cholesterol, HDL-C, low density lipoprotein cholesterol (LDL-C), and serum creatinine per standard NHANES protocols [18]. The estimated glomerular filtration rate (eGFR) was calculated using the Chronic Kidney Disease Epidemiology Collaboration (CKD-EPI) equation based on serum creatinine, age, sex, and race.

### 2.6. Statistical Analysis

Participants were categorized into four mutually exclusive groups based on the presence or absence of SCMI and insulin resistance (IR): those with low METS-IR values and no SCMI (reference group), those with high METS-IR values only, those with SCMI only, and those with both high METS-IR values and SCMI.

Baseline characteristics were compared across these groups. Continuous variables were summarized as means and standard deviations or medians and interquartile ranges, depending on their distribution, and compared using ANOVA or Kruskal–Wallis tests. Categorical variables were summarized as frequencies and percentages and compared using chi-square tests.

We specified two covariate-adjustment sets for all regression models. Model 1 included demographics and socioeconomic factors: age, sex, race/ethnicity, education, and household income. Model 2 extended Model 1 by additionally adjusting for smoking status, hypertension, total cholesterol, anti-hypertensive medication use, lipid-lowering medication use, estimated glomerular filtration rate (eGFR), and physical activity.

We evaluated the cross-sectional association between the METS-IR and SCMI using multivariable logistic regression, with SCMI as the dependent variable and the METS-IR assessed both as a continuous and categorical predictor. Associations were reported as adjusted odds ratios (ORs) with 95% confidence intervals (CIs).

CV mortality was analyzed using Cox proportional hazards regression. The proportional hazards assumption was formally tested using Schoenfeld residuals, and no significant violations were observed. The association between the METS-IR, SCMI, and cardiovascular death was then assessed across the four exposure groups, using the group with low METS-IR values and no SCMI as the reference. Two models were constructed: Model 1 adjusted for demographic variables including age, sex, race/ethnicity, income, and education level; Model 2 further adjusted for clinical and behavioral risk factors including hypertension, smoking status, physical activity, total cholesterol, estimated glomerular filtration rate, and use of anti-hypertensive and lipid-lowering medications. We additionally fit Cox models including a multiplicative SCMI × high METS-IR term and obtained *p* values for interactions.

We also examined whether the joint effects of SCMI and the METS-IR differed across key subgroups using fully adjusted Cox models. Subgroups were defined by sex, race (Black vs. non-Black), age (<65 vs. ≥65 years), smoking status (never vs. ever), BMI (<30 vs. ≥30 kg/m^2^), and annual household income (<$20,000 vs. ≥$20,000). For each subgroup, we estimated hazard ratios for the four SCMI + METS-IR categories within each level of the subgroup and tested for effect modification by each subgroup by adding cross-product terms between the four-level SCMI + METS-IR variable and the subgroup variable in the overall model.

A two-sided *p*-value of <0.05 was considered statistically significant for the main effects. However, for testing interaction terms, we pre-specified a *p*-value threshold of 0.1 to identify potential interactions, acknowledging that tests for interaction are often underpowered and require a less stringent threshold than main effect testing [22,23].

All analyses were performed using SAS version 9.4 (SAS Institute Inc., Cary, NC, USA) and the R statistical computing environment (version 4.1.3; http://www.r-project.org) (accessed on 5 February 2025).

## 3. Results

### 3.1. Study Population Characteristics

A total of 6079 participants were included in the analytic cohort (mean age 55.8 ± 10.7 years; 52.4% women). Of these, 1520 participants (25.0%) had a high METS-IR value (≥75th percentile) and 1474 (24.2%) had electrocardiogram-defined subclinical myocardial injury (SCMI).

Baseline characteristics stratified by METS-IR and SCMI status are shown in Table 1. Compared with participants with low METS-IR values and no SCMI, those with both high METS-IR values and SCMI were older and had substantially higher body mass index and systolic blood pressure values, as well as higher total cholesterol and LDL cholesterol levels. This group also had the highest prevalence of diabetes mellitus and the greatest use of antihypertensive medications. All between-group comparisons were statistically significant.

### 3.2. Association Between METS-IR and Subclinical Myocardial Injury

In cross-sectional analyses, higher METS-IR values were associated with greater odds of SCMI (Table 2). In fully adjusted logistic regression models, each 1-standard-deviation increase in the METS-IR was associated with 22% higher odds of SCMI. When examined categorically, participants with high METS-IR values had 60% higher odds of SCMI compared with those with low METS-IR values. All associations were highly statistically significant (all *p* < 0.0001).

### 3.3. METS-IR, SCMI, and Cardiovascular Mortality

Over a median follow-up of 18.8 years (interquartile range 15.6–20.8), 561 cardiovascular deaths occurred, corresponding to an event rate of 9.2%. Cardiovascular mortality was more frequent among participants with high versus low METS-IR values (10.8% vs. 8.5%) and among those with versus without SCMI (14.4% vs. 7.5%).

In fully adjusted Cox proportional hazards models, high METS-IR values were associated with an approximately 33% higher risk of cardiovascular mortality compared with low METS-IR values, while SCMI was associated with a roughly 44% higher risk compared with no SCMI (Table 3).

### 3.4. Joint Association of METS-IR and SCMI with Cardiovascular Mortality

When the METS-IR and SCMI were examined jointly, a graded increase in cardiovascular mortality risk was observed. The cumulative incidence of cardiovascular death ranged from 7.3% among participants with neither high METS-IR values nor SCMI to 17.0% among those with both conditions.

Compared with the reference group (low METS-IR values and no SCMI), cardiovascular mortality risk was modestly increased among participants with SCMI alone and was highest among those with both SCMI and high METS-IR values. In fully adjusted models, the coexistence of high METS-IR values and SCMI was associated with nearly a twofold higher risk of cardiovascular mortality (hazard ratio 1.98; 95% confidence interval 1.53–2.56).

**Figure 2:**
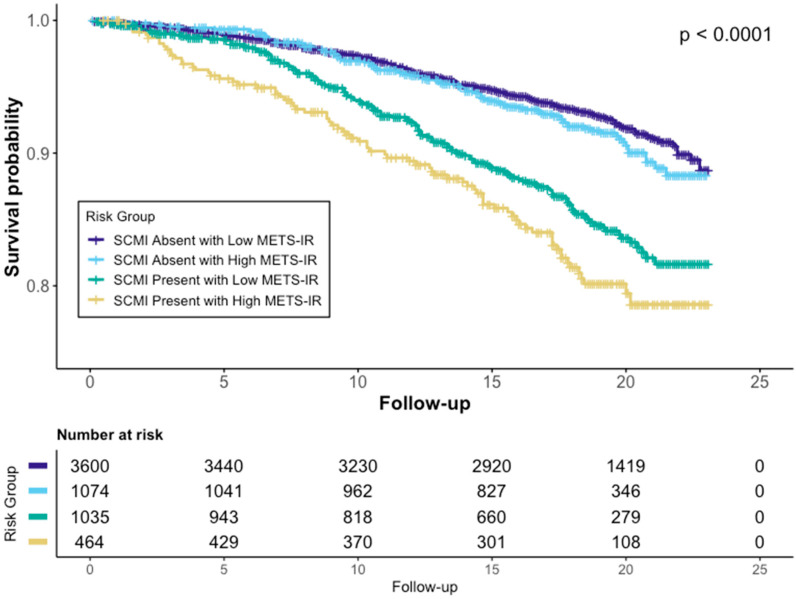
Kaplan–Meier Survival Curves for Cardiovascular Mortality by METS-IR and SCMI Status. SCMI, subclinical myocardial injury; METS-IR, Metabolic Score for Insulin Resistance.

Kaplan–Meier survival curves demonstrated progressively lower cardiovascular survival across the four METS-IR/SCMI groups, with the poorest survival observed in participants with both high METS-IR values and SCMI (Figure 2). Formal tests for statistical interaction between the METS-IR and SCMI were not significant (interaction *p* = 0.54 in Model 1 and *p* = 0.34 in Model 2).

### 3.5. Subgroup Analyses

Subgroup analyses stratified by sex, race, age, smoking status, body mass index, and income are presented in Table 4. Across most subgroups, participants with both high METS-IR values and SCMI consistently exhibited the highest cardiovascular mortality risk.

Evidence of effect modification was observed only for smoking status (interaction *p* = 0.091). Among ever-smokers, the combination of high METS-IR values and SCMI was associated with a more pronounced increase in cardiovascular mortality risk, whereas associations among never-smokers were weaker and not statistically significant. No significant effect modification was detected for sex, race, age, body mass index, or income (all interaction *p*-values > 0.10).

## 4. Discussion

In this analysis from the NHANES III study we examined the association of the METS-IR with SCMI as well as their independent and combined associations with CV mortality. Our analysis revealed several key findings: First, we observed a strong association between the METS-IR and SCMI, emphasizing the role of IR in subclinical CVD and suggesting a potential mechanistic link between metabolic dysfunction and cardiac injury. Second, both higher METS-IR values and the presence of SCMI independently predicted increased long-term CV mortality, reinforcing their importance as predictors of adverse outcomes. Finally, individuals with both high METS-IR values and SCMI exhibited the highest risk, with nearly a twofold increase in hazard compared with those without either condition.

Prior research has independently established both IR and subclinical cardiac injury as predictors of adverse cardiovascular outcomes [4,13]. However, our study uniquely contributes to the existing body of knowledge by explicitly investigating their combined effect, highlighting the clinical importance of concurrent metabolic and cardiac abnormalities. The METS-IR, a reliable and practical index derived from readily available clinical measurements, has shown robust predictive capacity for cardiometabolic outcomes, including ischemic heart disease, heart failure, and stroke [14,15,16,24]. Our findings align with these studies by demonstrating that high METS-IR values independently predict a 33% increased risk of CV mortality, reinforcing the METS-IR’s utility in identifying individuals at heightened cardiovascular risk.

The pathophysiological basis underlying the association between IR and cardiovascular risk likely involves a complex interaction between metabolic dysregulation, systemic inflammation, and endothelial dysfunction, all of which contribute to the atherosclerotic process [25,26]. Elevated IR, reflected by higher METS-IR scores, may directly promote a pro-atherogenic environment, leading to structural changes in the coronary vasculature and increasing vulnerability to myocardial injury even in the absence of clinical symptoms [5,27,28]. Our cross-sectional analyses confirm that each unit increase in the METS-IR significantly raises the odds of SCMI, indicating that IR may play an early and critical role in myocardial pathology.

SCMI is a strong independent predictor of mortality as previously shown in multiple studies [3,4]. In this analysis, SCMI was associated with a 44% higher risk of CV death after adjusting for potential confounders. This is consistent with earlier research demonstrating that SCMI significantly increases mortality risk even among asymptomatic individuals [3]. The ability to detect SCMI on a routine ECG emphasizes its clinical value for identifying asymptomatic individuals at risk of future CV mortality.

A critical finding of our study is the markedly elevated risk in participants with both high METS-IR values and SCMI. This co-exposure conferred nearly double the risk of CV mortality compared with those without either condition. From a clinical standpoint, our findings suggest that the combined use of routine 12-lead ECG and the METS-IR may provide a practical approach to cardiovascular risk stratification in individuals without clinically apparent CVD. The markedly elevated risk observed among individuals with both high METS-IR values and SCMI in our study suggests that metabolic dysfunction may potentiate myocardial vulnerability, identifying a subgroup at particularly high long-term cardiovascular risk. Because both ECG and the METS-IR rely on inexpensive and widely available measurements, their combined assessment could help identify high-risk individuals who may otherwise be classified as low or intermediate risk using conventional approaches.

Importantly, the METS-IR offers important advantages for both clinical and epidemiologic applications. Gold-standard methods such as the hyperinsulinemic–euglycemic clamp provide precise quantification of insulin sensitivity but are impractical for routine care or large population studies [11]. Traditional surrogate indices, including fasting insulin and the Homeostasis Model Assessment of Insulin Resistance (HOMA-IR), require insulin measurements that are not universally available and may be subject to assay variability. In contrast, the METS-IR is derived exclusively from routinely collected clinical parameters and has been shown to outperform the HOMA-IR and other alternative measures of insulin resistance in predicting cardiometabolic outcomes [11,13]. This supports the potential role of the METS-IR as a scalable and clinically accessible marker of insulin resistance for cardiovascular risk stratification, particularly when combined with measures of early myocardial damage.

In subgroup analyses, the joint association of the METS-IR and SCMI with cardiovascular mortality was generally consistent across sex, race, age, BMI, and income, with no clear evidence of effect modification by these factors. In contrast, smoking status showed possible effect modification, with a borderline interaction (*p* for interaction = 0.091). These findings suggest that the adverse impact of combined metabolic dysfunction and subclinical cardiac injury may be amplified in individuals with a history of smoking, a group already at elevated cardiovascular risk [29]. Given the limited power for interaction testing and the borderline *p* value, these results should be interpreted cautiously but may help generate hypotheses for future studies targeting high-risk subgroups of smokers.

Our study has several notable strengths, including its large, nationally representative sample and comprehensive adjustment for demographic and clinical confounders. However, it is also subject to limitations. First, the observational design precludes causal inference due to potential residual confounding and bias. In particular, the exclusion of baseline CVD relied on self-reported data, which is subject to recall and misclassification bias. Despite this limitation, both predictors used in the analysis (SCMI and the METS-IR) have been extensively linked to CV mortality in previous studies with diverse populations [3,4,6,10,13,30,31,32,33,34]. Second, although the METS-IR is a validated surrogate for insulin resistance, it remains an indirect measure, and direct assessment methods such as the hyperinsulinemic–euglycemic clamp were not utilized. Third, the baseline data in the NHANES III were collected between 1988 and 1994, which may limit generalizability to contemporary populations and current clinical practice. CV mortality has declined substantially over the past three decades, underscoring the need for validation in more recent cohorts. Lastly, while SCMI identification through ECG has predictive validity, additional imaging modalities like cardiac MRI or echocardiography could further refine myocardial injury detection.

## 5. Conclusions

Our study demonstrates that the METS-IR is associated with greater risk of SCMI, and that both the METS-IR and SCMI independently predict increased CV mortality in individuals free of overt CVD. These findings emphasize the importance of integrated cardiometabolic assessment, highlighting the potential of the METS-IR and ECG-based screening to identify individuals at substantial cardiovascular risk, thereby guiding targeted preventive interventions.

## Figures and Tables

**Table 1:** Baseline Characteristics among 6079 Participants in NHANES III Study by Metabolic Score for Insulin Resistance and Subclinical Myocardial Injury Status.

Variable	Overall (*n* = 6079)	Low METS-IR with Absent SCMI (*n* = 3548)	High METS-IR with Absent SCMI (*n* = 1057)	Low METS-IR with Present SCMI (*n* = 1011)	High METS-IR with Present SCMI (*n* = 463)	*p*-Value
Age, years; mean (SD)	55.79 (10.66)	54.84 (10.64)	54.36 (10.17)	59.71 (10.49)	57.39 (9.99)	<0.001
Female, *n* (%)	3185 (52.4)	1884 (53.1)	573 (54.2)	524 (51.8)	202 (43.6)	<0.001
Race/Ethnicity, *n* (%)						<0.001
Non-Hispanic White	2793 (45.2)	1644 (46.3)	413 (39.1)	502 (49.7)	198 (42.8)	
Non-Hispanic Black	1498 (24.3)	838 (23.6)	237 (22.4)	275 (27.2)	118 (25.5)	
Mexican American	1616 (26.2)	878 (24.7)	367 (34.7)	208 (20.6)	138 (29.8)	
Other	266 (4.3)	188 (5.3)	40 (3.8)	26 (2.6)	9 (1.9)	
Education ≥ High School, *n* (%)	3475 (57.2%)	2135 (60.2%)	540 (51.1%)	531 (52.5%)	235 (50.8%)	<0.001
Income < $20,000 per year	2599 (42.1)	1355 (38.2)	478 (45.2)	479 (47.4)	226 (48.8)	<0.001
Ever Smoker, *n* (%)	3473 (56.3)	1963 (55.3)	531 (50.2)	645 (63.8)	281 (60.7)	<0.001
BMI, mean (SD)	27.95 (5.58)	25.82 (3.51)	34.52 (5.21)	25.58 (3.76)	34.56 (5.73)	<0.001
LDL-C, mg/dL; mean (SD)	136.54 (40.94)	135.05 (42.22)	137.47 (38.51)	138.73 (38.01)	140.58 (41.53)	0.136
HDL-C, mg/dL; mean (SD)	51.07 (16.38)	54.50 (16.08)	40.84 (10.76)	54.90 (17.43)	39.72 (11.37)	<0.001
Lipid-lowering Medications, *n* (%)	258 (4.2)	131 (3.7)	53 (5.0)	48 (4.7)	22 (4.8)	0.162
Total Cholesterol, mg/dL; mean (SD)	222.23 (44.00)	220.12 (42.56)	223.26 (45.07)	224.69 (45.50)	230.62 (47.66)	<0.001
SBP, mmHg; mean (SD)	130.78 (26.82)	128.16 (25.71)	133.77 (36.83)	133.52 (20.02)	137.90 (18.42)	<0.001
DBP, mmHg; mean (SD)	77.63 (25.16)	76.97 (25.55)	80.28 (36.54)	76.39 (10.33)	79.64 (10.92)	<0.001
eGFR, (mL/min/1.73 m^2^); mean (SD)	71.30 (17.30)	73.14 (14.27)	73.09 (15.85)	69.20 (16.23)	70.24 (17.05)	<0.001
Anti-hypertension Medications, *n* (%)	1243 (20.1)	531 (15.0)	278 (26.3)	239 (23.6)	172 (37.1)	<0.001
Diabetes Mellitus, *n* (%)	955 (15.5)	301 (8.5)	287 (27.2)	128 (12.7)	154 (33.3)	<0.001
Physically Active, *n* (%)	4245 (68.8)	2560 (72.2)	671 (63.5)	668 (66.1)	289 (62.4)	<0.001

NHANES III, the Third National Health and Nutrition Examination Survey; METS-IR, metabolic score for insulin resistance; SCMI, subclinical myocardial injury; SD, standard deviation; BMI, body mass index; LDL-C, low-density lipoprotein cholesterol; HDL-C, high-density lipoprotein cholesterol; SBP, systolic blood pressure; DBP, diastolic blood pressure; mg/dL, milligrams per deciliter; mmHg, millimeters of mercury; eGFR, estimated glomerular filtration rate. Low METS-IR: <75th percentile; high METS-IR: ≥75th percentile (cohort-based). SCMI was defined as a cardiac infarction injury score (CIIS) ≥ 10. Categorical variables were compared using the chi-square test; continuous variables were compared using ANOVA or the Kruskal–Wallis test, as appropriate. A *p*-value < 0.05 was considered statistically significant.

**Table 2:** Cross-Sectional Association Between Metabolic Score for Insulin Resistance and Subclinical Myocardial Injury.

	Model 1		Model 2	
	OR (95% CI)	*p*-Value	OR (95% CI)	*p*-Value
Continuous				
METS-IR (per SD) *	1.22 (1.15–1.29)	<0.0001	1.20 (1.13–1.27)	<0.0001
Categorical				
Low METS-IR	Reference	--	Reference	--
High METS-IR	1.60 (1.40–1.82)	<0.0001	1.58 (1.31–1.90)	<0.0001

METS-IR, Metabolic Score for Insulin Resistance; OR, odds ratio; CI, confidence interval. Low METS-IR: <75th percentile; high METS-IR: ≥75th percentile (cohort-based). * Per 1-standard-deviation increase in METS-IR (SD = 10.95 units). Model 1 adjusted for age, sex, race, income, and education level. Model 2 adjusted for Model 1 and smoking status, hypertension, total cholesterol, anti-hypertensive medications, lipid lowering medications, estimated glomerular filtration rate, and physical activity.

**Table 3:** Separate and Joint Associations of METS-IR and SCMI with Cardiovascular Mortality.

Exposure Categories		No. Events (%)	Model 1		Model 2	
			HR (95%CI)	*p*-Value	HR (95%CI)	*p*-Value
Separate Associations						
METS-IR Status	Low METS-IR	394 (8.5%)	Ref.	--	Ref.	--
	High METS-IR	167 (10.8)	1.44 (1.20–1.73)	<0.001	1.33 (1.11–1.60)	<0.003
SCMI Status	SCMI Absent	352 (7.5)	Ref.	--	Ref.	--
	SCMI Present	211 (14.4)	1.63 (1.37–1.94)	<0.001	1.44 (1.21–1.72)	<0.001
Joint Associations						
METS-IR & SCMI Categories	SCMI Absent with Low METS-IR	264 (7.3)	Ref.	--	Ref.	--
	SCMI Absent with High METS-IR	88 (8.2)	1.23 (0.97–1.57)	0.088	1.15 (0.90–1.47)	0.277
	SCMI Present with Low METS-IR	130 (12.6)	1.44 (1.17–1.79)	<0.001	1.29 (1.04–1.60)	0.020
	SCMI Present with High METS-IR	79 (17.0)	2.37 (1.84–3.06)	<0.001	1.98 (1.53–2.56)	<0.001

SCMI, subclinical myocardial injury; METS-IR, Metabolic Score for Insulin Resistance. Low METSIR: <75th percentile; high METSIR: ≥75th percentile (cohort-based). Model 1 adjusted for age, sex, race, income, and education level. Model 2 adjusted for Model 1 and smoking status, hypertension, total cholesterol, anti-hypertensive medications, lipid lowering medications, estimated glomerular filtration rate, and physical activity.

**Table 4:** Association of Metabolic Score for Insulin Resistance and Subclinical Myocardial Injury with Cardiovascular Mortality in Subgroups.

Subgroups	METS-IR	SCMI	No. Events (%)	Event Rate (/1000 PY)	Hazard Ratio (95% CI)	*p*-Value	Interaction *p*-Value
Men	Low	Absent	139 (8.2%)	4.77	Reference	--	0.569
	Low	Present	72 (14.5%)	10.11	1.25 (0.931–1.67)	0.139	
	High	Absent	42 (8.6%)	4.96	1.02 (0.673–1.54)	0.931	
	High	Present	46 (17.6%)	11.07	1.28 (0.764–2.13)	0.350	
Women	Low	Absent	125 (6.6%)	3.61	Reference	--	
	Low	Present	58 (10.9%)	6.61	1.32 (0.962–1.81)	0.085	
	High	Absent	46 (7.9%)	4.52	1.39 (0.895–2.15)	0.143	
	High	Present	33 (16.2%)	10.59	1.56 (0.901–2.71)	0.112	
Blacks	Low	Absent	72 (8.1%)	4.64	Reference	--	0.807
	Low	Present	37 (12.8%)	8.55	1.01 (0.666–1.52)	0.977	
	High	Absent	22 (8.8%)	5.22	1.31 (0.698–2.46)	0.401	
	High	Present	16 (12.9%)	8.35	1.18 (0.54–2.56)	0.683	
Non-Blacks	Low	Absent	192 (7.1%)	3.98	Reference	--	
	Low	Present	93 (12.6%)	8.03	1.40 (1.08–1.8)	0.010	
	High	Absent	66 (8%)	4.58	1.15 (0.816–1.63)	0.422	
	High	Present	63 (18.4%)	11.76	1.38 (0.9–2.13)	0.138	
Age < 65 years	Low	Absent	110 (4%)	2.14	Reference	--	0.965
	Low	Present	39 (6.3%)	3.64	1.19 (0.819–1.72)	0.364	
	High	Absent	51 (6%)	3.31	1.43 (0.939–2.18)	0.096	
	High	Present	41 (12.3%)	7.32	1.44 (0.828–2.52)	0.195	
Age ≥ 65 years	Low	Absent	154 (18.3%)	12.47	Reference	--	
	Low	Present	91 (22.1%)	17.56	1.31 (1–1.71)	0.048	
	High	Absent	37 (16.5%)	11.54	0.95 (0.613–1.46)	0.801	
	High	Present	38 (28.8%)	22.76	1.36 (0.798–2.3)	0.260	
Never Smoker	Low	Absent	100 (6.2%)	3.39	Reference	--	0.091
	Low	Present	41 (11%)	6.56	1.23 (0.85–1.78)	0.272	
	High	Absent	46 (8.6%)	4.9	1.53 (0.961–2.44)	0.073	
	High	Present	22 (12%)	7.21	0.96 (0.507–1.8)	0.887	
Ever Smoker	Low	Absent	164 (8.2%)	4.79	Reference	--	
	Low	Present	89 (13.6%)	9.22	1.31 (1–1.7)	0.049	
	High	Absent	42 (7.8%)	4.54	0.91 (0.609–1.36)	0.651	
	High	Present	57 (20.1%)	13.51	1.74 (1.08–2.82)	0.024	
BMI < 30	Low	Absent	220 (7%)	3.95	Reference	--	0.260
	Low	Present	117 (12.9%)	8.49	1.39 (1.11–1.76)	0.005	
	High	Absent	10 (5.7%)	3.3	0.95 (0.497–1.81)	0.873	
	High	Present	17 (18.3%)	11.78	1.25 (0.55–2.82)	0.599	
BMI ≥ 30	Low	Absent	44 (9.9%)	5.45	Reference	--	
	Low	Present	13 (10.5%)	6.13	0.79 (0.421–1.47)	0.451	
	High	Absent	78 (8.7%)	5.0	1.00 (0.663–1.5)	0.984	
	High	Present	62 (16.6%)	10.64	2.28 (1.12–4.63)	0.023	
Annual Income <$20,000	Low	Absent	151 (10.9%)	6.55	Reference	--	0.703
	Low	Present	81 (16.6%)	11.91	1.25 (0.942–1.65)	0.123	
	High	Absent	51 (10.4%)	6.19	1.15 (0.774–1.7)	0.496	
	High	Present	47 (20.5%)	14.34	1.48 (0.91–2.4)	0.114	
Annual Income ≥$20,000	Low	Absent	113 (5.1%)	2.78	Reference	--	
	Low	Present	49 (9.1%)	5.38	1.28 (0.915–1.8)	0.148	
	High	Absent	37 (6.3%)	3.56	1.12 (0.701–1.78)	0.645	
	High	Present	32 (13.5%)	8.01	1.33 (0.732–2.4)	0.351	

SCMI, subclinical myocardial injury; METS-IR, Metabolic Score for Insulin Resistance, BMI, body mass index; PY, person-years of follow-up. Low METS-IR: <75th percentile; high METS-IR: ≥75th percentile (cohort-based). Model adjusted for age, sex, race and education level, hypertension, total cholesterol, anti-hypertensive medications, lipid lowering medications, smoking, estimated glomerular filtration rate, and physical activity.

## Data Availability

The data analyzed in this study are publicly available from the National Health and Nutrition Examination Survey (NHANES), conducted by the Centers for Disease Control and Prevention (CDC). NHANES datasets, including laboratory, examination, questionnaire, and electrocardiogram data, can be accessed at https://www.cdc.gov/nchs/nhanes/ (accessed on 5 February 2025). Linked mortality follow-up data are available through the National Center for Health Statistics (NCHS) Linked Mortality Files. No new data were created for this study. Derived variables and analytic code used to generate the results are available from the corresponding author upon reasonable request.

## Abbreviations

The following abbreviations are used in this manuscript:

CI	Confidence Interval
CIIS	Cardiac Infarction Injury Score
CKD-EPI	Chronic Kidney Disease Epidemiology Collaboration
CV	Cardiovascular
CVD	Cardiovascular Disease
ECG	Electrocardiogram
eGFR	Estimated Glomerular Filtration Rate
HDL-C	High-Density Lipoprotein Cholesterol
HOMA-IR	Homeostasis Model Assessment of Insulin Resistance
HR	Hazard Ratio
ICD-10	International Classification of Diseases, 10th Revision
IR	Insulin Resistance
LDL-C	Low-Density Lipoprotein Cholesterol
METS-IR	Metabolic Score for Insulin Resistance
NDI	National Death Index
NHANES III	Third National Health and Nutrition Examination Survey
OR	Odds Ratio
SCMI	Subclinical Myocardial Injury
